# The liver steatosis severity and lipid characteristics in primary biliary cholangitis

**DOI:** 10.1186/s12876-021-01974-4

**Published:** 2021-10-22

**Authors:** Yuan Zhang, Xing Hu, Jing Chang, Jie Chen, Xue Han, Tieying Zhang, Jianjun Shen, Ning Shang, Jing Han, Hui Wang, Weiwei Kang, Fankun Meng

**Affiliations:** 1 Capital Medical University, No 8, Xitoutiao, Youanmenwai, Fengtai District, Beijing, 100069 China; 2 Capital Medical University, Beijing, China; 3 Hepatology Immunology Department, Beijing You’an Hospital, Capital Medical University, Beijing, China; 4 Function Diagnosis Department, Handan Infectious Disease Hospital, Handan, China; 5 Infection Center Department, Beijing You’an Hospital, Capital Medical University, Beijing, China; 6 Hepatology and Nephrology Department, Beijing You’an Hospital, Capital Medical University, Beijing, China

**Keywords:** Primary biliary cholangitis, Controlled attenuation parameter, Liver steatosis, High-density lipoprotein cholesterol, Dyslipidemia

## Abstract

**Background:**

Patients with primary biliary cholangitis (PBC) often have comorbid dyslipidemia, and determining the degree of hepatic steatosis can help predict the risk of cardiovascular events in PBC patients. The aim of our study was to analyze the characteristics of lipid distribution and the degree of hepatic steatosis in PBC.

**Methods:**

We retrospectively analyzed 479 cases of PBC, chronic hepatitis B (CHB), chronic hepatitis C (CHC), non-alcoholic fatty liver disease (NAFLD), and healthy subjects (Normal) diagnosed by liver biopsy or definitive clinical diagnosis. Controlled attenuation parameter (CAP) values were applied to assess the degree of steatosis of the liver, and lipid levels were also compared in the five cohorts.

**Results:**

We found that among the five groups of subjects, the PBC group had the lowest CAP values (*P* < 0.001), and the high-density lipoprotein cholesterol (HDL-C) level in the PBC group was higher than normal, CHC and CHB group (*P* = 0.004, *P* = 0.033, *P* < 0.001, respectively).In the multivariate linear analysis, only BMI (β = 1.280, *P* = 0.028), ALP (β = − 0.064, *P* = 0.012), TBA (β = − 0.126, *P* = 0.020), TG (β = 12.520, *P* = 0.000), HDL-C (β = − 11.338, *P* = 0.001) and LDL-C (β = 7.012, *P* = 0.002) were independent predictors of CAP.

**Conclusions:**

Among PBC, CHB, CHC, NAFLD and healthy subjects, PBC had the lowest degree of hepatic steatosis and higher HDL-C levels, all of which were found to be protective factors against atherosclerosis and cardiovascular risk and would provide a valuable reference for the risk of developing cardiovascular events in PBC patients.

**Supplementary Information:**

The online version contains supplementary material available at 10.1186/s12876-021-01974-4.

## Background

Primary biliary cholangitis (PBC) is a chronic, progressive cholestatic autoimmune disease that tends to occur in women above 40 years of age, the male-to-female ratio being 1:10 [1], and 76% of patients with the condition often have comorbid dyslipidemia [2]. Although a high proportion of patients with PBC have dyslipidemia, the increased risk of atherosclerosis and cardiovascular events is controversial. It has been suggested that the low risk of cardiovascular events in PBC may be associated with low visceral fat content [3], while other studies found that patients with progressive PBC and moderate hypercholesterolemia had a risk of developing cardiovascular disease [2]. When lipid metabolism is abnormal, there is an increased degree of hepatic steatosis [4]. Liver steatosis is associated with increased risk of cardiovascular events [5]. Thus, understanding the degree of hepatic steatosis is important in determining the risk of cardiovascular events in patients with PBC.

Liver steatosis is widespread in chronic liver disease with different etiologies [6–8]. Currently, chronic hepatitis B (CHB), chronic hepatitis C (CHC), and nonalcoholic fatty liver disease (NAFLD) are the most common chronic liver diseases (CLD) [9]. Liver biopsy is currently the gold standard for evaluating liver steatosis [10]. However, because it is invasive, it entails a high risk of bleeding. Many studies have found that transient elastography (TE)-based controlled attenuation parameter (CAP) value can accurately assess the severity of liver steatosis [6], Specifically, CAP value increases with the severity of liver steatosis and can be used to diagnose steatosis of > 5% [11]. In the present study, we retrospectively analyzed a PBC cohort to explore the characteristics of lipid distribution in patients with PBC, and used CAP value to determine the differences liver steatosis severity between PBC and other CLD.

## Methods

### Design of the study and patients

**Fig. 1:**
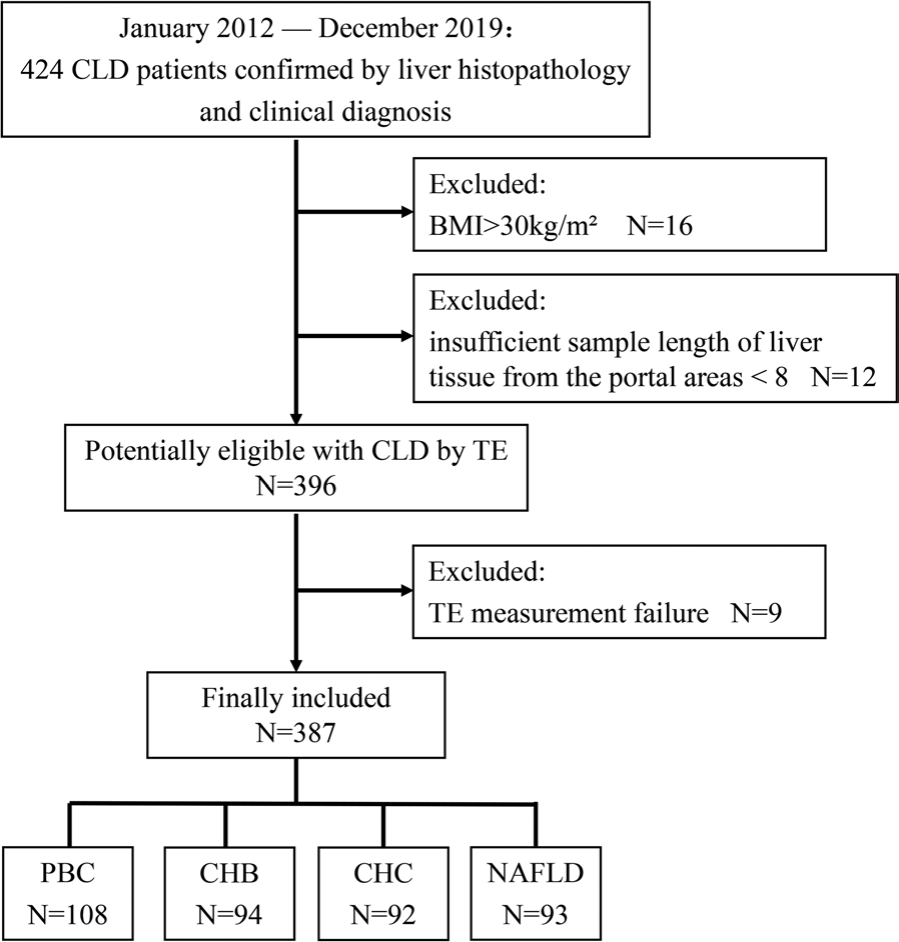
Study flow chart

In this retrospective study, we selected 424 patients who were diagnosed with PBC, CHB, CHC, or NAFLD by either liver biopsy or clinical examination between January 2012 and December 2019. 37 patients were excluded from the study, of which 12 had an insufficient sample length of liver tissue from the portal areas < 8, 9 had TE measurement failure, and 16 had a body mass index (BMI) > 30 kg/m^2^. Finally, 387 patients with chronic liver disease and 92 healthy volunteers were enrolled. In all patients, PBC had been confirmed by liver histopathology, and 212 patients had CHB, CHC, or NAFLD, as confirmed by liver histopathology (88.3%, 65.2%, 74.1%, respectively). Diagnostic criteria for all included patients were in accordance with current guidelines [1, 7, 12, 13]. The inclusion criteria for healthy volunteers were normal transaminase levels, while the exclusion criteria were hyperlipidemia, disease history in the heart, lung, or liver, malignancy, excessive alcohol consumption and long-term medication use. The BMI of all patients with chronic liver disease and healthy volunteers was ≤ 30 kg/m^2^ (Fig. 1). In this retrospective study, the requirement for informed consent was waived, and approval was granted by the ethical committee of the Beijing Youan Hospital, Capital Medical University. The study protocol confirmed to the ethical guidelines of the Declaration of Helsinki.

Two investigators were responsible for collecting the demographic data (sex, age, BMI) and clinical data of the subjects, as well as their biochemical and hematological data within 1 week of liver biopsy, including aspartate aminotransferase (AST), alanine aminotransferase (ALT), total bilirubin (TBil), direct bilirubin (DBil), alkaline phosphatase (ALP), gamma-glutamyl transferase (GGT), total bile acid (TBA), total cholesterol (TC), triglyceride(TG), high-density lipoprotein cholesterol (HDL-C), and low-density lipoprotein cholesterol (LDL-C).

### Transient elastography

During examination, CAP values were simultaneously measured using the Fibroscan 502 with M probe (Echosens Inc., France), which was placed vertically at the right 7th and 8th intercostal space at the proposed liver biopsy site. Multiple continuous measurements were taken. The operating standards were as follows: 10 successful tests, success rate of 60% and above, quartile deviation for liver elasticity lower than 30% of the median [14]. The operator was an ultrasound physician with 10 years’ experience who had passed Fibroscan training and examination.

### Liver biopsy

Liver biopsy was carried out on 320 patients with chronic liver disease. The sample length was 1.5–2.0 cm incorporating ≥ 8 portal areas. Each pathological smear was diagnosed by an experienced pathologist who had been blinded to the clinical and imaging examination results.

### Statistical analysis

Descriptive statistics were described using frequency and percentage for categorical data, mean and standard deviation for normally distributed continuous data, and median and interquartile range for non-normally distributed continuous data to summarize the demographic, clinical, and blood lipid characteristics of the PBC, CHC, CHB, NAFLD, and healthy control groups. One-way analysis of variance was used to compare normally distributed continuous data, and the least significant difference t-test was used for pairwise intra-group comparison. The Kruskal–Wallis H test was used for inter-group and intra-group pairwise comparison of non-normally distributed quantitative data. The chi-square test was used to compare categorical data. Multiple linear regression analysis was performed to identify independent predictors of CAP as the continuous dependent variable. As candidate risk factors, we selected sex, age, BMI, ALT, TBiL, ALP, GGT, TBA, TC, HDL-C and LDL-C. A *P* value < 0.05 was considered statistically significant. Kappa test was used to analyze the consistency between CAP value and pathological biopsy results to determine the degree of hepatic steatosis. IBM’s SPSS Statistics, version 24 (IBM Corp., Armonk, NY) and GraphPad Prism V.6.0 (San Diego, CA, USA) was used for analyses and graph preparation.

## Results

### Study population

A total of 479 subjects (91.9% females) were included in this cross-sectional study: 108 with PBC, 94 with CHB, 92 with CHC, 93 with NAFLD, and 92 healthy volunteers. The median age of all subjects was 52 (rang 26–70), and the median BMI was 25 kg/m^2^ (rang 17–30). The results of liver histopathological steatosis grading in all PBC patients were as follows: S0: 102 (< 10%), S1:5 (10–33%), S2:1 (34–66%), S3:0 (> 66%) [15]. All subjects received CAP measurements. Table 1 and Additional file 1: Table 1 detail the clinical and biological characteristics and CAP values of the different groups.

**Table 1:** Clinical and biological characteristics of subjects of five groups

	PBC (n = 108)	Normal (n = 92)	CHC (n = 92)	CHB (n = 94)	NAFLD (n = 93)	*P* value
Male gender	8 (7.4%)	8 (8.7%)	8 (8.7%)	8 (8.5%)	7 (7.5%)	0.995
Age (years)	52 (27–69)	52 (26–68)	52 (27–70)	51 (28–68)	52 (27–69)	0.989
BMI (kg/m^2^)	24.0 (18.0–30.0)	24.0 (17.0–30.0)	24.0 (19.0–30.0)	25.0 (18.0–30.0)	26.0 (18.0–30.0)	0.041
CAP (dB/m)	205.5 ± 34.7	221.1 ± 34.6	243.0 ± 35.7	228.0 ± 32.6	290.4 ± 39.1	< 0.001****
ALT (IU/L)	36.1 (8.0–920.0)	17.5 (3.0–40.0)	20.5 (5.0–1134.0)	23.5 (6.0–929.0)	52.0 (9.0–739.0)	< 0.001****
AST (IU/L)	46.5 (16.0–845.0)	20.0 (10.0–34.0)	24.5 (11.0–514.0)	23.0 (10.0–464.0)	52.0 (15.0–268.0)	< 0.001****
TBil (IU/L)	17.4 (6.7–443.0)	12.5 (6.4–21.0)	15.1 (6.8–109.5)	14.7 (5.9–146.0)	12.9 (6.4–193.5)	< 0.001****
DBil (IU/L)	5.1 (0.8–193.0)	4.2 (1.9–8.4)	5.1 (1.8–47.8)	4.4 (0.6–112.9)	4.0 (1.0–134.3)	0.002***
ALP (IU/L)	142.0 (26.0–1100.0)	71.5 (30.0–189.0)	76.5 (40.0–196.0)	70.0 (37.0–201.0)	87.0 (41.0–206.0)	< 0.001****
GGT (IU/L)	120.0 (9.0–1429.0)	17.0 (8.0–68.0)	20.0 (8.0–286.0)	19.5 (8.0–517.0)	52.5 (10.0–835.0)	< 0.001****
TBA (IU/L)	15.3 (1.3–408.8)	2.6 (0.6–28.7)	3.9 (0.8–73.9)	4.7 (0.8–182.3)	5.4 (1.0–71.3)	< 0.001****
TC (IU/L)	5.0 (1.0–14.0)	4.5 (2.9–5.2)	4.3 (1.0–7.0)	4.6 (2.5–7.7)	5.1 (2.0–7.0)	< 0.001****
TG (IU/L)	1.2 (0.5–7.4)	1.1 (0.5–1.7)	1.0 (0.4–4.4)	1.0 (0.4–6.8)	1.9 (0.6–15.1)	< 0.001****
HDL-C (IU/L)	1.5 (0.2–6.2)	1.3 (0.7–2.7)	1.3 (0.5–2.9)	1.4 (0.2–6.1)	1.2 (0.3–5.7)	< 0.001****
LDL-C (IU/L)	2.6 (0.8–6.2)	2.7 (1.0–3.9)	2.6 (0.6–5.3)	2.8 (1.1–5.9)	3.2 (0.8–5.5)	< 0.001****

Data are shown as mean ± SD (range) or median (range)

BMI, body mass index; CAP, controlled attenuation parameter; AST, aspartate aminotransferase; ALT, alanine aminotransferase; TBil, total bilirubin; DBil, direct bilirubin; ALP, alkaline phosphatase; GGT, gamma-glutamyl transferase; TBA, total bile acid; TC, total cholesterol; TG, triglyceride; HDL-C, high-density lipoproteincholesterol; LDL-C, Low-density lipoprotein cholesterol; PBC, primary biliary cholangitis; CHB, chronic hepatitis B; CHC, chronic hepatitis C; NAFLD, nonalcoholic fatty liver diseas e

*P < 0.05; **P < 0.01; ***P < 0.005; ****P < 0.001

### Analysis of lipid characteristics

**Fig. 2:**
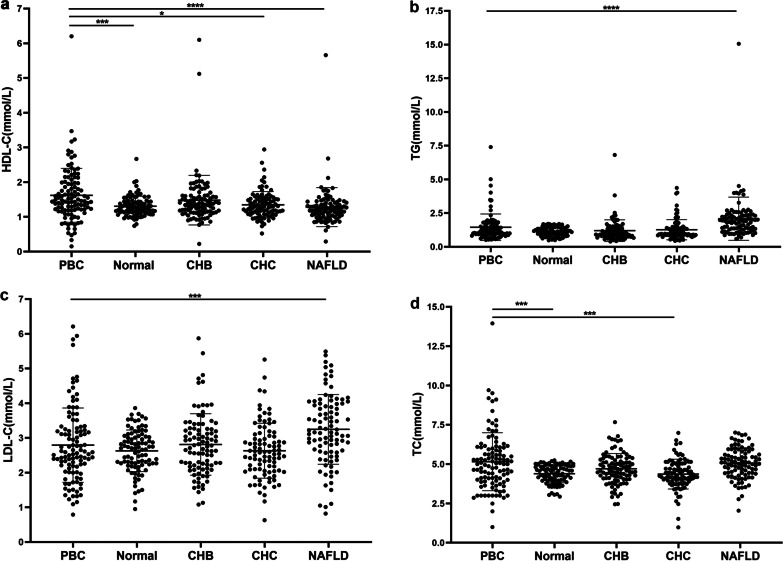
Scatter plot of lipid of health adults and patients with hepatic disease of different causes. Kruskal Wallis H test was used to compare the values of HDL-C, TG, LDL-C, and TC between five groups of subjects, and only results of pairwise comparisons between PBC patients and the other four groups are shown by the horizontal lines and asterisks. **a** HDL-C, **b** TG, **c** LDL-C, **d** TC. *P < 0.05; **P < 0.01; ***P < 0.005; and ****P < 0.001. Abbreviation: HDL-C, high-density lipoprotein cholesterol; TG, triglyceride; LDL-C, low-density lipoprotein cholesterol; TC, total cholesterol; PBC, primary biliary cholangitis; CHB, chronic hepatitis B; CHC, chronic hepatitis C; NAFLD, nonalcoholic fatty liver disease

Among 387 patients with chronic liver disease, 75 (69.4%) PBC, 41 (44.6%) CHC, 47 (50%) CHB, and 84 (90.3%) NAFLD had dyslipidemia. To assess the differences in lipids between PBC and other common chronic liver diseases and normal subjects, we analyzed lipid levels in five groups: 90 (83.3%) PBC, 81 (88.0%) normal, 77 (83.7%) CHC, 82 (87.2%) CHB, and 69 (74.1%) NAFLD groups had normal HDL-C. In the PBC group, the lipid levels were higher than those of the other four groups. Although the proportion of normal HDL-C was not significantly different between the PBC group and the other four groups (*P* = 0.088), the HDL-C level in the PBC group was higher than that in the normal group, CHC group and NAFLD group (1.5 mmol/L vs 1.3 mmol/L,1.3 mmol/L,1.2 mmol/L, *P* = 0.004, *P* = 0.033, *P* < 0.001, respectively), which was not different from the CHB group (Fig. 2a). TG and LDL-C in the PBC group were only lower than those in the NAFLD group (*P* < 0.001, *P* = 0.001, respectively) and not different with other groups (Fig. 2b, c), and TC in the PBC group was higher than that in the normal and CHC groups (*P* = 0.002, all), which was not different from the CHC and NAFLD groups (Fig. 2d). These results showed that the HDL-C and TC of PBC were relatively higher than those of the other groups.

### Liver steatosis assessed by CAP values

**Fig. 3:**
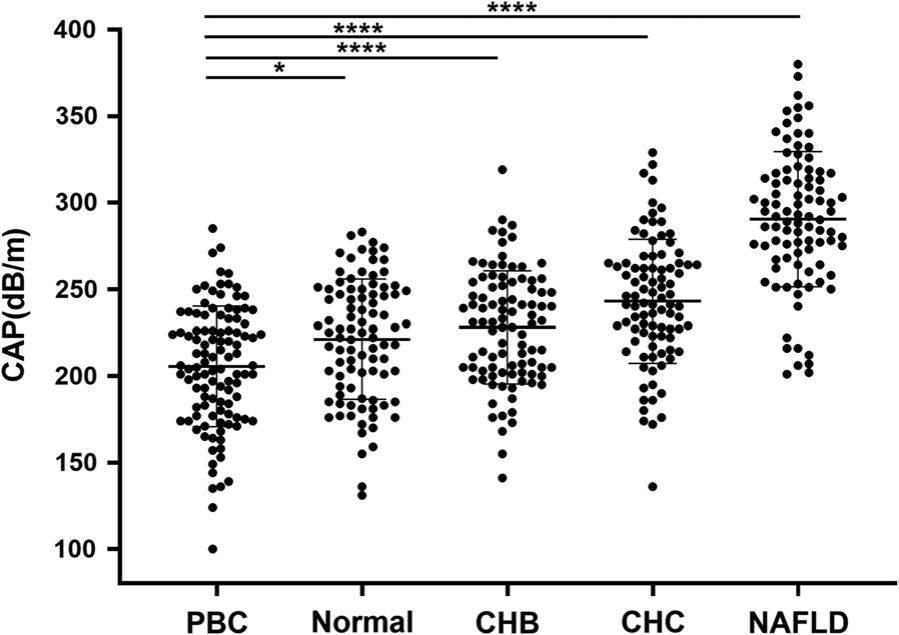
Scatter plot of the CAP values of health adults and patients with hepatic disease of different causes. Kruskal Wallis H test was used to compare the values of CAP between five groups of subjects, and only results of pairwise comparisons between PBC patients and the other four groups are shown by the horizontal lines and asterisks. *P < 0.05; **P < 0.01; ***P < 0.005; and ****P < 0.001. Abbreviation: CAP, controlled attenuation parameter; PBC, primary biliary cholangitis; CHB, chronic hepatitis B; CHC, chronic hepatitis C; NAFLD, nonalcoholic fatty liver disease

We assessed the degree of hepatic steatosis in PBC patients by analyzing the differences in liver CAP values between PBC and the other four groups. The mean CAP values and standard deviations of the patients with PBC, healthy subjects, and patients with CHB, CHC, or NAFLD were 205.5 ± 34.7 dB/m, 221.1 ± 34.6 dB/m, 228.0 ± 32.6 dB/m, 243.0 ± 35.7 dB/m, and 290.4 ± 39.1 dB/m, respectively. The lowest CAP values were found in the PBC group (all *P* < 0.05), the highest in the NAFLD group (all *P* < 0.001), and higher in the CHC group than in the normal and CHB groups (*P* < 0.001, *P* = 0.039, respectively), with no difference in CAP values between the normal and CHB groups; the above results indicate that the PBC group has the lowest degree of liver steatosis. The diagnostic cut-off values of 248 dB/m, 268 dB/m, and 280 dB/m were used as the diagnostic cut-off values for determining liver steatosis by CAP to analyze liver steatosis in patients with PBC [15]. In this study, 97patients with PBC had stage S0 (89.8%), 8 patients with S1 (7.4%), 2 patients with S2 (1.9%), and 1 patient with S3 (0.9%). This set of data reflects that 89.8% of PBC patients with liver steatosis are S0. Using liver histopathological findings as the gold standard, 107 patients with PBC were included, and the consistency of CAP values in determining the degree of hepatic steatosis in PBC was good according to the Kappa test (K = 0.734, *P* = 0.000) (one S0 stage PBC patient with a CAP value of 285 dB/m had liver tissue specimens full of dilated hepatic sinusoids with severe venous reflux, and this case was considered to affect the CAP value on the assessment of hepatic steatosis, so it was not included). Figure 3 shows the CAP values in the PBC group compared with those of the healthy subjects, as well as with those in the CHB, CHC, and NAFLD groups.

### Univariate and multivariate analysis for predictors of CAP

BMI, ALP, GGT, TBA, TG, HDL-C and LDL-C were associated with CAP in univariate analysis. However, only BMI (β = 1.280, *P* = 0.028), ALP (β = − 0.064, *P* = 0.012), TBA (β = − 0.126, *P* = 0.020), TG (β = 12.520, *P* = 0.000), HDL-C (β = − 11.338, *P* = 0.001) and LDL-C (β = 7.012, *P* = 0.002) independently predicted CAP on multivariate analysis (Table 2).

**Table2:** Univariate and multivariate linear regression analysis to identify factors influencing CAP value

Variable	Univariate analysis			Multivariable analysis		
	β coefficient	SE	*P* value	β coefficient	SE	*P* value
Male gender	7.754	7.643	0.311	–	–	–
Age (years)	0.109	0.222	0.623	–	–	–
BMI (kg/m^2^)	1.446	0.638	0.024	1.280	0.581	0.028
ALT (IU/L)	− 0.009	0.020	0.658	–	–	–
TBil (IU/L)	− 0.049	0.075	0.519	–	–	–
ALP (IU/L)	− 0.078	0.018	0.000	− 0.064	0.025	0.012
GGT (IU/L)	− 0.031	0.012	0.010	0.000	0.017	0.993
TBA (IU/L)	− 0.198	0.053	0.000	− 0.126	0.054	0.020
TC (IU/L)	1.118	1.728	0.518	–	–	–
TG (IU/L)	13.453	1.923	0.000	12.520	1.939	0.000
HDL-C(IU/L)	− 16.475	3.426	0.000	− 11.338	3.469	0.001
LDL-C(IU/L)	8.841	2.247	0.000	7.012	2.199	0.002

BMI, body mass index; ALT, alanine aminotransferase; TBil, total bilirubin; ALP, alkaline phosphatase; GGT, gamma-glutamyl transferase; TBA, total bile acid; TC, total cholesterol; TG, triglyceride; HDL-C, high-density lipoproteincholesterol; LDL-C, Low-density lipoprotein cholesterol

## Discussion

In the present study, we retrospectively analyzed CAP values and blood lipid levels in patients with a definitive diagnosis of PBC, CHB, CHC, or NAFLD, as well as in healthy controls, over a 8-year period. The novelty of the study is that, for the first time, CAP values were applied to determine the difference in liver steatosis between PBC and other common CLD, and for the first time, PBC was compared with lipids from other CLD, and we concluded that patients with PBC have low liver steatosis along with high HDL-C levels, Our findings may provide an informative basis for the risk of cardiovascular events in PBC.

Dyslipidemia is an important global problem [16]. Elevated TG, TC, LDL and reduced HDL-C are typical features of NAFLD [17, 18], which is consistent with our results. In the whole cohort, lipids in patients with PBC were not significantly different from those in patients with CHB. Siagris et al. demonstrated lower TC, LDL-C, and HDL-C in hepatitis C virus (HCV)-infected patients compared with the normal population [19]. Wong et al. [20] pointed out that in the CHB population, there was a significant reduction in TC and TG and no significant difference in HDL-C and LDL-C compared with the HBsAg-negative population. Our study showed no significant differences in lipids between CHC, CHB, and normal populations, which may be because the age, sex, and geographic region of the matched cases in this study differed from previous studies. In our study, HDL-C and TC levels were elevated in patients with PBC, and the mechanism of their elevation has been confirmed in previous studies [21, 22]. We demonstrated that HDL-C levels in PBC were not only higher than in the NAFLD and CHC groups, but also higher than in the normal population. The reduced HDL-C levels in NAFLD, the reduction is attributed to impairment of mature HDL during the conversion of newly-synthesized HDL to mature HDL [23], Indeed, impaired HDL maturation is an independent risk factor for predicting NAFLD atherosclerosis [24]. This shows the important role of HDL-C levels in predicting atherosclerotic events. HDL-C levels are elevated in patients with PBC, and LpA1 (lipoprotein a1), the main protein of HDL, promotes cholesterol efflux from adipocytes [25], and inhibits the accumulation of TC in the vessel wall, thereby reducing atherosclerosis. On the other hand, HDL increases the expression of lipocalin mRNA in partially differentiated adipocytes through the phosphatidylinositol-3 kinase (PI3K) pathway and elevates the concentration of plasma lipocalin [26], and the elevated level of lipocalin inhibits the conversion of macrophages to foam cells and resists the formation of atherosclerosis [27]. It has been demonstrated that elevated HDL-C concentrations lead to a reduced incidence of atherosclerosis in patients with PBC [28].

CAP can accurately predict lipid content in CLD [6, 29]; they are not affected by fibrosis or necrosis [30] and their accuracy does not vary with disease etiology [8]. In our study, NAFLD had the highest CAP value, followed by CHC, and then CHB had a higher CAP value than the normal group. There is no doubt that the CAP value is highest in the NAFLD group, Because of NAFLD is a clinicopathological syndrome characterized by diffuse hepatocyte macrofollicular lipodystrophy. In patients with CHC, the HCV core protein induces hepatocyte steatosis [31], and HCV infection is positively correlated with metabolic syndrome (MetS) [32]. Hepatitis B virus (HBV) infection seems to have a protective effect on steatosis progression [33], and HBV infection is positively correlated with MetS [33]. Thus, the effect of etiology on liver steatosis can be seen in both CHC and CHB groups, with some studies suggesting a lower prevalence of fatty liver in CHB than in CHC [34], and our study confirms a lower degree of liver steatosis in the CHB group than in the CHC group. Our study shows that PBC patients have the lowest degree of hepatic steatosis not only among the common chronic liver diseases, but also lower than healthy subjects.

It has been suggested that dyslipidemia is abnormal in PBC without a concomitant increase in hepatic steatosis, which is attributed to fibroblast growth factor 19 (FGF19), which has enhanced expression in PBC patients, and FGF19 induces a decrease in mitochondrial acetyl coenzyme A carboxylase-2 (AAC2), which in turn promotes free fatty acid oxidation; inhibit insulin-related fatty acid synthesis in hepatocytes by suppressing the activity of sterol regulatory element binding protein 1C and elevated SHP expression [35], and reduce hepatic fat accumulation and plasma triglyceride levels. Other studies have suggested that the dysbiosis of intestinal flora in PBC patients and the disturbance of hepatic bile acid circulation can cause steatorrhea, which impairs fat absorption in PBC patients [36, 37], and these factors may lead to reduced fat deposition in the liver. When PBC patients with total bilirubin levels above 4.5 mg/dL all suffer from severe steatorrhea [37], that would mean that all 108 PBC patients in our study had impaired fat absorption. It has also been proposed that elevated lipocalin levels in PBC patients inhibit hepatic gluconeogenesis, resist hepatic fat accumulation, are anti-inflammatory, enhance insulin sensitivity, and reduce insulin resistance [38], thereby reducing hepatic steatosis [39].

In the multivariate linear analysis, we found that BMI, ALP, TBA, TG, HDL-C and LDL-C were independent predictors of CAP. CAP values were not influenced by elevated transaminases and bilirubin, but were affected by ALP and TBA levels. Reshetnyak VI proposed that the elevated TC in PBC is a compensatory response of the body to cholestasis in order to neutralize excess bile acids [37], and this compensatory response rationalizes the anomaly of elevated TC levels and decreased CAP values in patients with PBC. There is evidence that patients with PBC are at risk of death due to atherosclerosis, but the mortality rate from the development of atherosclerosis does not differ from the normal population [40]. Our view is that the combined low degree of hepatic steatosis and high HDL-C levels in PBC are both protective factors against atherosclerosis and cardiovascular risk, and we hypothesize that patients with PBC have a lower risk of developing cardiovascular events.

The present study had some limitations. Firstly, it was a retrospective analysis carried out in a single center. Secondly, because age and sex have an effect on PBC onset, there were fewer patients with CHC, CHB, and NAFLD confirmed by liver histopathology than patients with PBC over the 8-year study period. Lastly, the causes of high HDL-C levels and liver steatosis low severity in PBC are complex, and we did not explore this in depth due to the limitations of the retrospective study. In our study, we did not have data on the occurrence of atherosclerosis and cardiovascular events in PBC patients, but in future studies, we will increase the sample size and further investigate the correlation between HDL-C levels, hepatic lipid metabolism mechanisms and the occurrence of atherosclerosis and cardiovascular events in PBC patients.

In conclusion, the present study revealed a low level of liver steatosis in PBC patients, which further confirms the high level of HDL-C. This is an encouraging finding and provides strong support that PBC patients are co-morbidly hyperlipidemic without an increased incidence of atherosclerosis and cardiovascular events. That said, the mechanism remains unclear and requires further attention and research.

## Supplementary Information

**Additional file 1: Table 1.** Blood lipid characteristics and CAP values of subjects of five groups.

## Data Availability

The datasets used and analyzed during the current study are available from the corresponding author on request.

## Abbreviations

PBC: Primary biliary cholangitis
CHB: Chronic hepatitis B
CHC: Chronic hepatitis C
NAFLD: Nonalcoholic fatty liver disease
CLD: Chronic liver disease
TE: Transient elastography
CAP: Controlled attenuation parameter
BMI: Body mass index
AST: Aspartate aminotransferase
ALT: Alanine aminotransferase
TBil: Total bilirubin
DBil: Direct bilirubin
ALP: Alkaline phosphatase
GGT: Gamma-glutamyl transferase
TBA: Total bile acid
TC: Total cholesterol
TG: Triglyceride
HDL-C: High-density lipoprotein cholesterol
LDL-C: Low-density lipoprotein cholesterol
HCV: Hepatitis C virus
LpA1: Lipoprotein a1
PI3K: Phosphatidylinositol-3 kinase
MetS: Metabolic syndrome
HBV: Hepatitis B virus
FGF19: Fibroblast growth factor 19
AAC2: Acetyl coenzyme A carboxylase-2
SHP: Small heterodimer partner
