# Supplementary material for: The liver steatosis severity and lipid characteristics in primary biliary cholangitis
Source: BMC Gastroenterol. 2021 Oct 22;21:395. doi: 10.1186/s12876-021-01974-4 (PMC8532358; doi:10.1186/s12876-021-01974-4)
Supplement: Supplementary file 1 — Additional file 1: Table 1. Blood lipid characteristics and CAP values of subjects of five groups. [file 12876_2021_1974_MOESM1_ESM.docx]

**Supplementary Information**

**Supplementary Table 1.** Blood lipid characteristics and CAP values of subjects of five groups.

|  | PBC  (n=108) | Normal  (n=92) | CHC  (n=92) | CHB  (n=94) | NAFLD  (n=93) | P  Value | P1  Value | P2  Value | P3  Value | P4  Value | P5  Value | P6  Value | P7  Value | P8  Value | P9  Value | P10  Value |
| --- | --- | --- | --- | --- | --- | --- | --- | --- | --- | --- | --- | --- | --- | --- | --- | --- |
| CAP (dB/m) | 205.5±34.7 | 221.1±34.6 | 243.0±35.7 | 228.0±32.6 | 290.4±39.1 | ＜0.001**^****^** | 0.019 | ＜0.001**^****^** | ＜0.001**^****^** | ＜0.001**^****^** | ＜0.001**^****^** | 1.000 | ＜0.001**^****^** | 0.039**^*^** | ＜0.001**^****^** | ＜0.001**^****^** |
| TC (IU/L) | 5.0  (1.0-14.0) | 4.5  (2.9-5.2) | 4.3  (1.0-7.0) | 4.6  (2.5-7.7) | 5.1  (2.0-7.0) | ＜0.001**^****^** | 0.002**^***^** | 0.002**^***^** | 1.000 | 1.000 | 1.000 | 0.283 | ＜0.001**^****^** | 0.249 | ＜0.001**^****^** | 0.111 |
| TG (IU/L) | 1.2  (0.5-7.4) | 1.1  (0.5-1.7) | 1.0  (0.4-4.4) | 1.0  (0.4-6.8) | 1.9  (0.6-15.1) | ＜0.001**^****^** | 1.000 | 0.623 | 0.102 | ＜0.001**^****^** | 1.000 | 1.000 | ＜0.001**^****^** | 1.000 | ＜0.001**^****^** | ＜0.001**^****^** |
| HDL-C (IU/L) | 1.5  (0.2-6.2) | 1.3  (0.7-2.7) | 1.3  (0.5-2.9) | 1.4  (0.2-6.1) | 1.2  (0.3-5.7) | ＜0.001**^****^** | 0.004**^***^** | 0.033**^*^** | 1.000 | ＜0.001**^****^** | 1.000 | 0.601 | 1.000 | 1.000 | 0.649 | 0.016**^*^** |
| LDL-C (IU/L) | 2.6  (0.8-6.2) | 2.7  (1.0-3.9) | 2.6  (0.6-5.3) | 2.8  (1.1-5.9) | 3.2  (0.8-5.5) | ＜0.001**^****^** | 1.000 | 1.000 | 1.000 | 0.001**^***^** | 1.000 | 1.000 | ＜0.001**^****^** | 1.000 | ＜0.001**^****^** | 0.006**^**^** |

Data are shown as mean ±SD (range)or median (range). P1, P value, PBC vs. Normal; P2, P value, PBC vs. CHC; P3, P value, PBC vs. CHB; P4，P value, PBC vs. NAFLD; P5，P value, Normal vs. CHC; P6，P value, Normal vs. CHB; P7，P value, Normal vs. NAFLD; P8，P value, CHC vs. CHB; P9，P value, CHC vs. NAFLD; P10，P value, CHB vs. NAFLD. *P<0.05; **P<0.01; ***P<0.005; and****P<0.001. Abbreviation: CAP, controlled attenuation parameter; TC, total [cholesterol](https://fanyi.so.com/?src=onebox#cholesterol); TG, [triglyceride](https://fanyi.so.com/?src=onebox#triglyceride); HDL-C, [high-density lipoprotein](https://fanyi.so.com/?src=onebox#high-density%20lipoprotein)[cholesterol](https://fanyi.so.com/?src=onebox#cholesterol); LDL-C, Low-density lipoprotein [cholesterol](https://fanyi.so.com/?src=onebox#cholesterol); PBC, primary biliary cholangitis; CHB, chronic hepatitis B; CHC, chronic hepatitis C; NAFLD, nonalcoholic fatty liver disease.
